# A Rare Occurrence of Geminated-Taloned Maxillary Lateral Incisor

**DOI:** 10.5005/jp-journals-10005-1151

**Published:** 2012-08-08

**Authors:** R Neeraja, Vizhi G Kayal

**Affiliations:** Reader, Department of Pedodontics, MR Ambedkar Dental College and Hospital, Bengaluru, Karnataka, India, e-mail: neeraja_pedo@yahoo.com; Reader, Department of Pedodontics, MR Ambedkar Dental College and Hospital, Bengaluru, Karnataka, India

**Keywords:** Talon's cusp, Gemination, Tooth abnormality, Anomaly

## Abstract

The talon cusp is a developmental anomaly characterized by the presence of an accessory cusp like structure projecting from the cingulum area of the anterior teeth. Gemination is an anomaly caused by a single tooth germ that attempted to divide during its development. These developmental anomalies may cause clinical problems including esthetic impairment, pain, caries and tooth crowding. Co-occurrence of two anomalies in a teeth is rare. This paper presents an unusual case of talon cusp on geminated permanent lateral incisor.

**How to cite this article:** Neeraja R, Kayal VG. A Rare Occurrence of Geminated-Taloned Maxillary Lateral Incisor. Int J Clin Pediatr Dent 2012;5(2):136-138.

## INTRODUCTION

The talon cusp, or dens evaginatus of anterior teeth, is a relatively rare developmental anomaly characterized by the presence of an accessory cusp like structure projecting from the cingulum area or cementoenamel junction of the maxillary or mandibular anterior teeth in both the primary and permanent dentition. This anomalous structure is composed of normal enamel and dentin and either has varying extensions of pulp tissue into it or is devoid of a pulp horn.^1^ The permanent dentition is affected more frequently than the primary dentition, and the anomaly is more common in males than in females.^2^ The talon cusp can occur as an isolated finding or in association with other dental anomalies, such as peg-shaped lateral incisor, agenesis or impacted canines, mesiodens, complex odontomes, megadont, dens evaginatus of posterior teeth, shovel-shaped incisors, dens invaginatus and exaggerated Carabelli cusp.^3^

Gemination is a developmental anomaly of form, which is recognized as an attempt by a single tooth germ to divide resulting in a large single tooth with bifid crown and usually common root and root canal.^1,4^ They are found more frequently in the primary than in permanent dentition, with a prevalence of approximately 1 and 0.1% respectively and more predilection in maxillary primary incisors and canine.^5^

A geminated permanent lateral incisor is an uncommon finding and the association of this geminated tooth with talons cusp is even rarer finding with only four previous case reports.^1,6-8^ Here we present a case of talon cusp on geminated permanent maxillary lateral incisor.

## CASE REPORT

A 10-year-old patient reported to our department complaining of large, unsightly maxillary lateral incisors. Family history was noncontributory. The patient appeared healthy and of normal physical development for his age. There was no reported history of orofacial trauma.

**Fig. 1:**
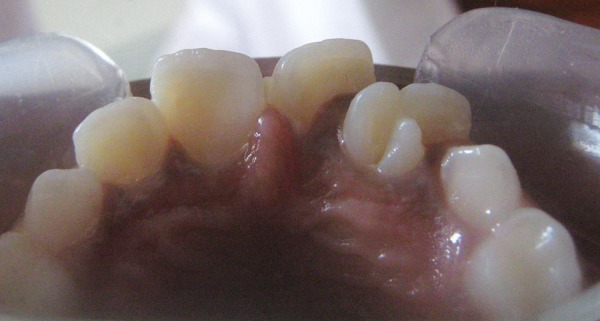
Palatal appearance of the geminated maxillary permanent lateral incisor showing the talon cusp

**Fig. 2:**
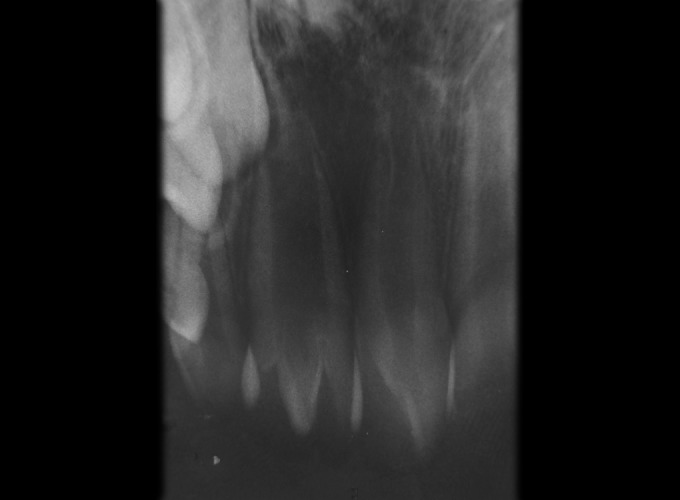
Periapical radiograph of the maxillary permanent lateral incisors

**Fig. 3:**
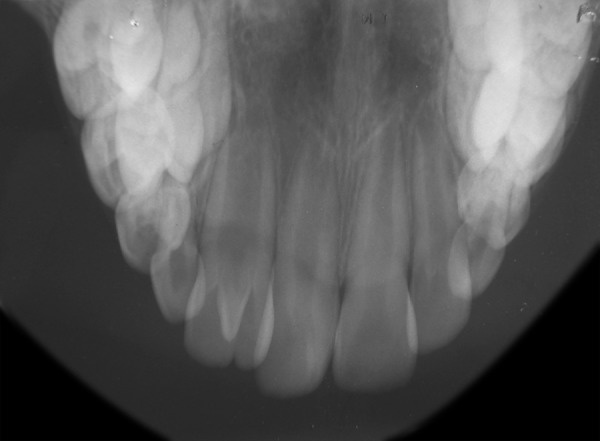
Occlusal radiograph of the maxillary permanent lateral incisors

Intraoral examination revealed mixed dentition and the occlusion was a class I molar relationship. The maxillary left central incisor had a large and bifid crown with accessory cusp on the palatal aspect. The mesiodistal crown diameter was 3 mm larger than the maxillary right lateral incisor. The crown of this tooth was partially split and had a notch on the incisal edge that extended labiolingually to the middle of the crown. On the palatal aspect, the crown exhibited a pronounced, well-defined accessory cusp extending from the cementoenamel junction to within 0.5 mm of the incisal edge. It was pyramidal in shape and located on the mesial half of the crown, with the tip of the cusp attached to the crown (Fig. 1). The cusp measured 6 mm in length (incisocervically), 4 mm in width (mesiodistally) and 3.5 mm in thickness (labiolingually). Deep developmental grooves were present at the junction of the accessory cusp and the palatal surface of the tooth. The affected tooth responded normally to electric and thermal pulp tests. A periapical and occlusal radiograph (Figs 2 and 3) showed a V-shaped radiopaque structure superimposed on the image of the affected crown, with the ‘V' pointing toward the incisal edge. The accessory cusp was outlined by two distinct white lines converging from the cervical area of the affected tooth toward the incisal edge. Pulp extension could be traced radiographically to the middle of the cusp. This tooth had a single enlarged pulp chamber, one root and bifid crown appearance. Based upon clinical and radiographic findings, a diagnosis of talon cusp on geminated tooth was made.

**Fig. 4:**
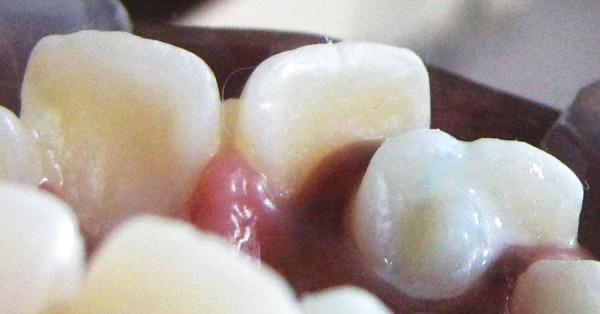
The restored grooves

The deep developmental grooves present at the junction of the talon and the palatal surface were restored with flowable composite, Tetric-N Flow (Ivoclar Vivadent) (Fig. 4).

## DISCUSSION

The etiology of talon cusp remains unknown, but it seems to have both genetic and environmental components.^9^ It is believed that the talon cusp originates during the morphodifferentiation stage of tooth development in which it may occur as a result of outward folding of inner enamel epithelial cells and transient focal hyperplasia of the peripheral cells of mesencymal dental papilla.^3^

The etiology of geminated teeth remains unknown. Spouge suggests that the condition may result from trauma to the developing tooth bud.^10^ Evidence from case history studies suggests that the anomaly exhibits a hereditary tendency, similar to that affecting the dental lamina and resulting in hyperdontia.^11^ The mode of inheritance is probably either autosomal recessive or dominant with very little penetrance.^12^ It appears that gemination is caused by complex interactions among a variety of genetic and environmental factors.

Even though mesiodens, microdontia, dens evaginatus on posterior teeth, shovel-shaped incisors, dens invaginatus or Carabelli tubercule overgrowth have been reported with talon cusp,^13^ cases of talon cusp associated with gemination are very rare. Gemination is often confused with fusion.^14^ Clinical-radiographic examination and history review will usually provide enough information to arrive at a definite diagnosis of gemination or fusion. Additionally, the determination of total tooth number in the arch may be helpful in distinguishing between fusion and gemination. In cases of fusion, when the anomalous tooth is counted as one, tooth count reveals a missing tooth, while in gemination there is a normal tooth count. However, distinguishing gemination and fusion by tooth count alone is not a definitive parameter in all cases because fusion can occur between a normal and a supernumary tooth.^6^ The patient described in this paper had the aforementioned features and therefore the anomaly affecting the maxillary left central incisor was diagnosed as gemination.

Early diagnosis of talon cusp is important and, in most cases, a definitive treatment is required. If the grooves are decayed, the lesion should be removed and the cavity restored. In case of premature contact and occlusal interference, the talon cusp should be reduced gradually on consecutive visits over 6- to 8-week intervals in order to allow time for deposition of reparative dentin for pulpal protection. In our case there was no interference with occlusion so the cusp was not reduced but the grooves were deep so it was restored to prevent carious involvement.

## CONCLUSION

Geminated-taloned teeth cause a variety of clinical problems that call for early diagnosis. So the clinician should be able to diagnose such a tooth and provide appropriate treatment at the right time.
